# Lack of Evidence from Studies of Soluble Protein Fragments that Knops Blood Group Polymorphisms in Complement Receptor-Type 1 Are Driven by Malaria

**DOI:** 10.1371/journal.pone.0034820

**Published:** 2012-04-10

**Authors:** Patience B. Tetteh-Quarcoo, Christoph Q. Schmidt, Wai-Hong Tham, Richard Hauhart, Haydyn D. T. Mertens, Arthur Rowe, John P. Atkinson, Alan F. Cowman, J. Alexandra Rowe, Paul N. Barlow

**Affiliations:** 1 The Institute of Structural and Molecular Biology, University of Edinburgh, Edinburgh, United Kingdom; 2 Walter and Eliza Hall Institute of Medical Research, Parkville, Victoria, Australia; 3 Division of Rheumatology, Department of Medicine, Washington University School of Medicine, St. Louis, Missouri, United States of America; 4 SAXS/WAXS Beamline, Australian Synchrotron, Clayton, Australia; 5 School of Biosciences, University of Nottingham, Sutton Bonington, Leicester, United Kingdom; 6 Institute of Immunology and Infection Research, University of Edinburgh, Edinburgh, United Kingdom; 7 School of Chemistry, University of Edinburgh, Edinburgh, United Kingdom; Bernhard Nocht Institute for Tropical Medicine, Germany

## Abstract

Complement receptor-type 1 (CR1, CD35) is the immune-adherence receptor, a complement regulator, and an erythroid receptor for *Plasmodium falciparum* during merozoite invasion and subsequent rosette formation involving parasitized and non-infected erythrocytes. The non-uniform geographical distribution of Knops blood group CR1 alleles *Sl1/2* and *McC^a/b^* may result from selective pressures exerted by differential exposure to infectious hazards. Here, four variant short recombinant versions of CR1 were produced and analyzed, focusing on complement control protein modules (CCPs) 15–25 of its ectodomain. These eleven modules encompass a region (CCPs 15–17) key to rosetting, opsonin recognition and complement regulation, as well as the Knops blood group polymorphisms in CCPs 24–25. All four CR1 15–25 variants were monomeric and had similar axial ratios. Modules 21 and 22, despite their double-length inter-modular linker, did not lie side-by-side so as to stabilize a bent-back architecture that would facilitate cooperation between key functional modules and Knops blood group antigens. Indeed, the four CR1 15–25 variants had virtually indistinguishable affinities for immobilized complement fragments C3b (*K*_D_ = 0.8–1.1 µM) and C4b (*K*_D_ = 5.0–5.3 µM). They were all equally good co-factors for factor I-catalysed cleavage of C3b and C4b, and they bound equally within a narrow affinity range, to immobilized C1q. No differences between the variants were observed in assays for inhibition of erythrocyte invasion by *P. falciparum* or for rosette disruption. Neither differences in complement-regulatory functionality, nor interactions with *P. falciparum* proteins tested here, appear to have driven the non-uniform geographic distribution of these alleles.

## Introduction

**Figure 1:**
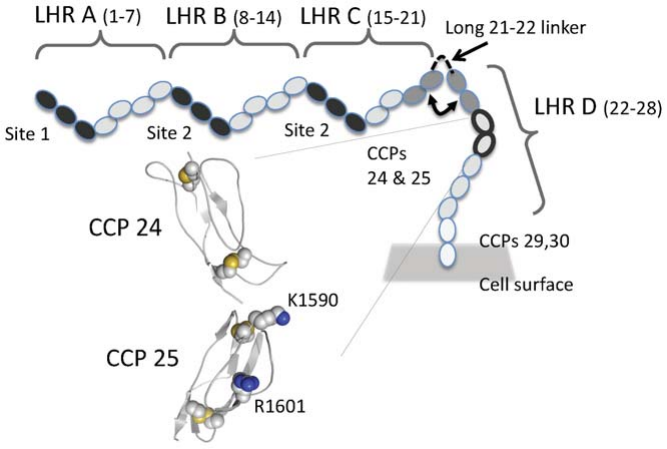
Schematic of CR1. (**A**) Main cartoon shows the 30 CCPs (ovals) in the ectodomain of the most common CR1 size-variant. Long-homologous repeats (LHRs) and positions of functional sites (dark grey) are indicated; the eight-residue linker between long-homologous repeats C and D is drawn as a dotted line along with neighbouring modules (grey); CCPs 24/25 wherein reside the *Sl1/2* and *McC^a/b^*-encoded antigens are also highlighted (thicker outline) and their modeled 3D structures [43] drawn as cartoons with K1590 and R1601 side chains shown as spheres.

The Swain-Langley (*Sl1/2*) and McCoy (*McC^a/b^*) Knops blood group antithetical antigen pairs lie within the erythrocyte-borne membrane glycoprotein, complement-receptor type 1 (CR1) (CD35) [1], [2]. Both the encoded amino acid sequence variations occur in the vicinity of the 25^th^ complement control protein (CCP) module (or short consensus repeat) out of the 30 CCP modules that constitute the ∼1800-residue N-terminal ectodomain of the most common size-variant of CR1 [3] (Fig. 1). The strikingly non-uniform geographical distribution of these alleles has been much discussed [4] [5]. Notably, both *McC^b^* (coding for E1590, *McC^a^* codes for K1590) and *Sl2* (coding for G1601, *Sl1* codes for R1601) are extremely rare in Caucasoids but common (prevalence of 45% and 80%, respectively) in individuals of African descent [6]. Such population differences probably arose due to selective pressure and it was hypothesized that the *Sl2* (and possibly *McC^b^*) alleles afford a survival advantage in the context of a geographically localized environmental hazard such as is posed by the malaria parasite *Plasmodium falciparum* [4], [5], [7]. Malaria is probably responsible for ∼1,100,000 deaths annually in Africa [8] but is largely absent from Europe and North America.

Erythroid CR1 is a ligand used by *P. falciparum* merozoites for sialic acid-independent invasion of red blood cells [9], [10], [11]. Moreover, CR1 on non-parasitized erythrocytes is a ligand for *P. falciparum* erythrocyte membrane protein 1 (PfEMP1) borne on infected erythrocytes; this interaction mediates formation of “rosettes”, which consist of clusters of infected and non-infected cells [12]. Rosette formation is associated with life-threatening forms of cerebral malaria [13]. Correlation between CR1 polymorphisms (*Sl2/Sl2 vs. Sl1/Sl1*) and resistance to severe malaria was reported in a western Kenyan population [14], but not in two studies of populations from The Gambia [15], [16].

Selective-pressure hypotheses require that *McC* and *Sl*-encoded variants diverge with respect to copy number expressed or to structure-function relationships; but this has not been investigated comprehensively. Complement receptor-type 1 [17] regulates classical and alternative pathways of the complement system. It is also the immune-adherence receptor, mediating transport by erythrocytes of particles opsonised with complement-activation specific fragments, C3b and C4b, to the primary lymphoid system for clearance [18]. Modules 1–3 (site 1), 8–10 (site 2) and 15–17 (a near-identical copy of site 2) are the first three modules of, respectively, the first, second and third (out of four), highly similar long-homologous repeats (A–D) accounting for the N-terminal 28 CCPs of the most common CR1 size variant (Fig. 1) [19]. These sites interact with opsonic C3b and C4b [20] and, to a lesser degree, the C3b breakdown product, iC3b, and are collectively crucial to the immune-clearance role of CR1. They also accelerate decay of C3 and C5 convertases, crucial multiple-subunit enzymes in the complement cascade. Moreover, site 2 is a cofactor for proteolytic cleavage of C3b and C4b [21], [22], [23], [24] by factor I that interrupts the complement cascade and generates ligands for other complement receptors linking complement to cellular and antibody-mediated immunity [25]. Importantly, site 1 also mediates interactions with Rh4 [26] vital for sialic acid-independent invasion by *P. falciparum* [11] while site 2 is a candidate for interaction with PfEMP1 and rosette formation [27].

It is intriguing that the Knops blood group antigens in CCPs 24–25 do not occupy known functional sites of the CR1 ectodomain. Residues encoded by *Sl1/2* and *McC^a/b^* lie within long-homologous repeat D, seven modules away (in the primary structure) from the nearest proven functional site (*i.e.* CCPs 15–17 or site 2 within long-homologous repeat C). Nonetheless, sites of sequence variation could be spatially apposed to one or more functional sites depending upon the overall architecture of CR1's long chain of CCPs [28], [29], [30], [31]. In particular, the uniquely long linking sequence of eight amino acid residues (compared to four residues on average) connecting CCPs 21 and 22 (*i.e.* between long-homologous repeats C and D, see Fig. 1) could represent a molecular hinge, enabling the modules of long-homologous repeat D to fold back onto long-homologous repeat C and hence influence the activity of the second copy of site 2.

Thus the possibility requires investigation that sequence variations in or near to CCP 25 influence C3b/C4b binding, complement-regulatory activity or binding to Rh4 or PfEMP1, despite being remote in the sequence from the primary sites of interaction. It is also possible that long-homologous repeat D possesses its own less well characterized or yet-to-be discovered binding sites for host or parasite proteins. For example, classical complement pathway protein C1q (part of the C1 complex that also includes the serine protease C1r and C1s) reportedly binds long-homologous repeat D of CR1 [32].

We prepared four eleven-CCP constructs, representing the *McC* and *Sl*-encoded CR1 variants, along with a set of shorter recombinant constructs encompassing modules at the border between long-homologous repeats C and D (CCP 21–CCP 22). We were thus able to show that Knops blood group variants in long-homologous repeat D neither modulate structure nor influence known functional activities of CR1 that might underlie selective pressure.

## Results

### A panel of truncations were designed for this study and produced in *P. pastoris*

**Figure 2:**
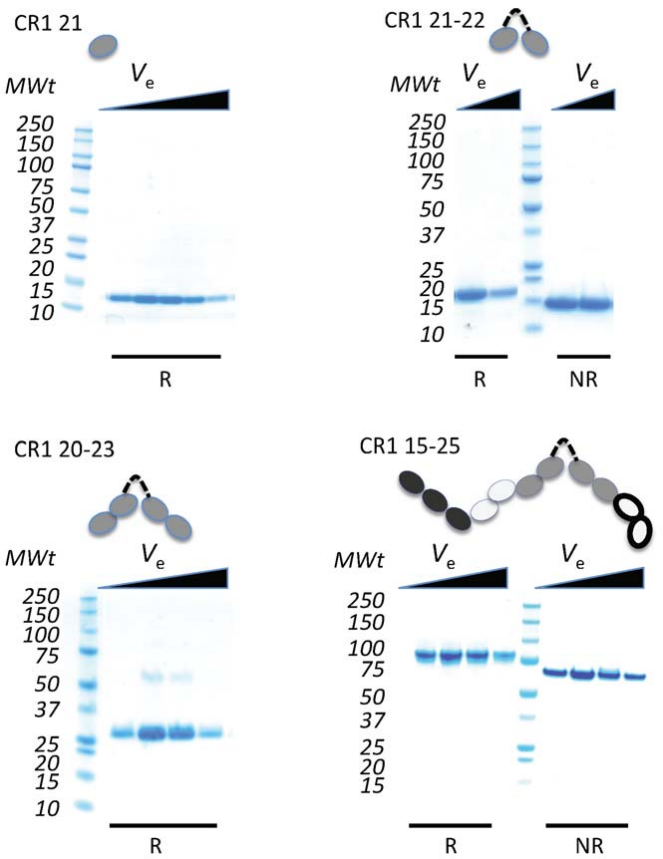
Recombinant CR1 truncations produced for the current study. The Coomassie-stained polyacrylamide gels show the results of electrophoresis of successive fractions (with increasing elution volumes, *V*_e_) from size-exclusion chromatography for CR1-21, CR1 21–22, CR1 20–23 and CR1 15–25 (the K1590,R1601 variant); reducing (R) conditions in some lanes would reveal products of intra-modular proteolytic “clipping” that would be held together by disulfide bonds under non-reducing (NR) conditions.

We made truncations of CR1 (Fig. 2) in the same strain of *Pichia pastoris* previously employed to produce biologically active full-length factor H (FH) (a CR1 homologue containing 20 CCPs) [33]. Recombinant CR1 15–25, *i.e.* CCPs 15–25, was designed as the minimal CR1 fragment spanning the second copy of site 2 (CCPs 15–17) and variant residues 1601 (*Sl1/2*) in CCP 25 and 1590 (*McC^a/b^*) in the linker between CCPs 24 and 25.

Producing the four variants of this eleven-module fragment - Glu^1590^/Gly^1601^ (EG, from *McC^b^/Sl2*), Lys^1590^/Arg^1601^ (KR, from *McC^a^/Sl1*, >98% prevalence in Caucasoids), Lys^1590^/Gly^1601^ (KG, from *McC^a^/Sl2*) and Glu^1590^/Arg^1601^ (ER, from *McC^b^/Sl1*) - in preference to an entire (*e.g.* 30-module) ectodomain of CR1, allowed us to focus on a single functional site. Moreover, unlike in the case of variants of CCPs 1–30, large amounts of material for quantitative measurements and biophysical characterisation could be produced. We also produced smaller fragments, CR1 21, CR1 21–22 and CR1 20–23 (Fig. 2), to investigate the potential of the double-length linker between CCPs 21 and 22 to promote a bent-back module chain, in which CCPs 24/25 lie close to CCPs 15–17. All proteins were secreted in good yields and purified (Fig. 2) using sequential ion-exchange and gel-filtration chromatographic steps.

### CCPs 21 and 22 of CR1 do not form a side-by-side interaction

**Figure 3:**
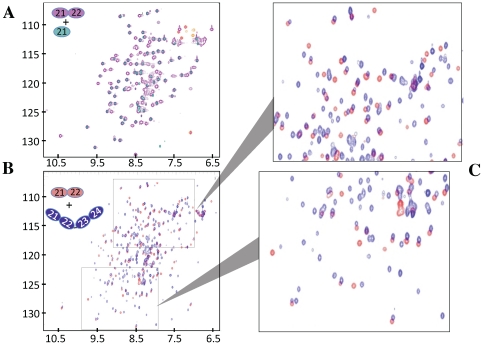
CCPs 21–24 do not exhibit extensive inter-modular contacts. (**A**) ^1^H,^15^N heteronuclear single quantum coherence (HSQC) NMR spectrum of CR1 21 overlaid on that of CR1 21–22. (**B**) An overlay of the HSQC spectra for CR1 21–22 and 20–23 with (**C**) blow-ups of two regions. Cross-peaks are color-coded according to the CR1 fragment of origin, as indicated. A dearth of chemical shift changes for CCP 21 and CCP 21–22 amides following attachment of neighbouring modules suggests chemical shift-perturbing inter-modular contacts are limited.

We compared ^1^H,^15^N-HSQC NMR spectra of CR1 21, CR1 21–22, and CR1 20–23 (Fig. 3). In these spectra each amide group produces a cross peak the position of which is determined by its ^15^N (*y*-axis) and ^1^H (*x*-axis) chemical shifts. The spectra were of high quality, featuring predominantly uniform line-widths and excellent chemical shift dispersion, showing that these proteins are properly folded. Nearly all cross peaks in the CR1 21 spectrum overlap with cross peaks in the CR1 21–22 spectrum (Fig. 3A), showing that attachment of CCP 22 to CCP 21 causes only minor perturbations of chemical shifts for amide groups in CCP 21. This implies that these modules share only a small inter-modular interface consistent with an end-to-end (rather than side-by-side) interaction. Likewise, many cross peaks in the partially assigned spectrum of CR1 21–22 match to cross peaks in the spectrum of CR1 20–23 (Figs. 3B,C); of those that do not, none corresponded to residues in the linker between CCPs 21 and 22; in fact most lie in module 22 (data not shown). These observations are consistent with CCPs 21 and 22 retaining their end-to-end disposition within the context of CR1 20–23.

**Figure 4:**
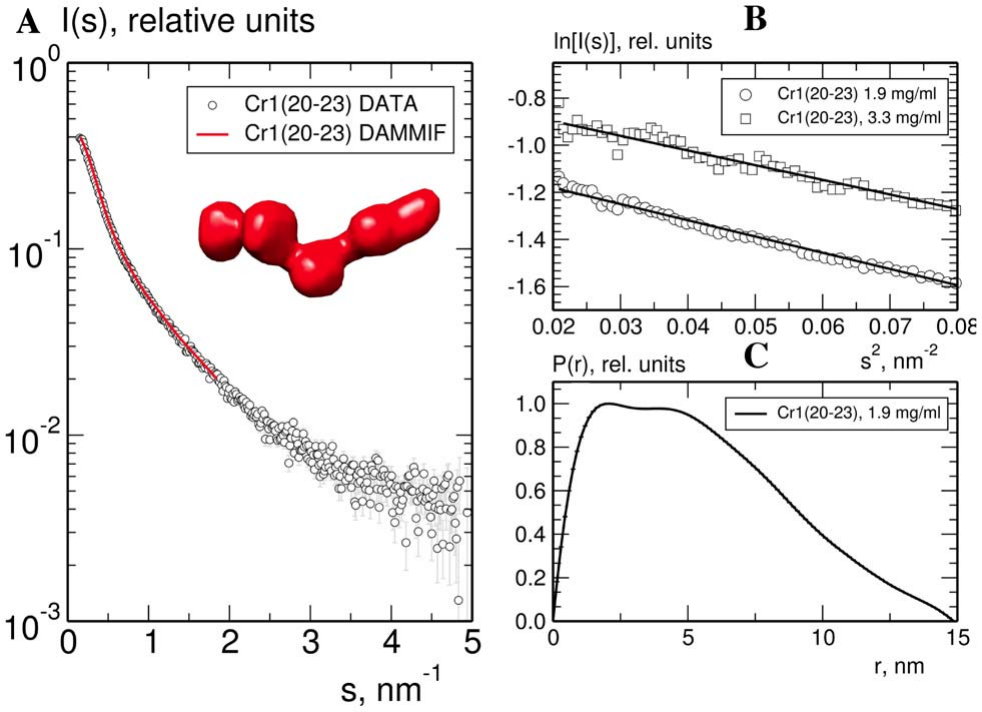
CCPs 21–24 do not exhibit extensive inter-modular contacts. (**A**) SAXS profile and fit of the DAMMIF *ab initio* model (red surface) to the CR1 20–23 data (1.9 mg.ml^−1^); (**B**) Guinier plot of the CR1 20–23 SAXS data at 1.9 mg.ml^−1^ (open circles) and 3.3 mg.ml^−1^ (open squares), showing no significant concentration dependence of SAXS parameters (plots displaced on the vertical axis for clarity); (**C**) Distance distribution function, *p(r)* for CR1 20–23.

To further investigate spatial arrangements of modules in CR1 20-23, samples were subjected to small-angle X-ray scattering (SAXS) generating high-quality data with no concentration dependency (Figs. 4A,B). The resulting DAMMIF *ab initio* model, with an excluded volume of 59 nm^3^ - consistent with that expected for a monomeric, properly folded, CR1 20–23 (29 Kd) - fitted well to the SAXS profile (discrepancy, χ = 1.5) (Fig. 4A). Both the model and the distance distribution, with a *D*_max_ of 15 nm (Fig. 4C), are fully consistent with a largely elongated arrangement of the four modules (see insert in (Fig. 4A) and cannot be reconciled with a stable bent-back conformation as was formed, for example, by the six CCPs at the centre of FH that had a *D*_max_ of 10.4 nm [34].

Thus, in CR1 20–23, the long linker between CCPs 21 and 22 (*i.e.* between long-homologous repeats C and D) does not induce a U-bend. Together with their immediate neighbours, these modules form a highly elongated structure. Extrapolating to a larger construct, CCPs 19 and 24 would be ∼15 nm apart, disfavouring apposition of CCPs 15–17 and CCPs 24–25. These results suggest that sequence variations in CCPs 24–25 (of long-homologous repeat D) have only local structural effects. To test this, analytical ultracentrifugation (AUC) was used to compare CR1 15–25 allotypic variants.

### Biophysical analysis reveals no differences in gross structures of variants

We subjected solutions of the CR1 15–25 allotypic variants to AUC and compared their sedimentation coefficients and axial ratios (Table 1). All had Svedberg values in the narrow range of 3.14–3.25, and calculated hydrodynamic radii in the range 5.55–5.80 nm, with no indication of any loss of folded structure or self-association over the concentration range (0.1–1 mg.ml^−1^) measured. These results are in good agreement with estimates of particles size distribution from dynamic light scattering (Fig. S1). Their calculated axial ratios were also similar, within experimental error, falling in the range of 5.6–6.6.

**Table 1:** AUC data for four CR1 15–25 variants.

Sample	mg/ml	*s* 1 (S)	*s(20,w)* [mean] (S)		*f/f_0_* SEDFIT	*f/f_0_ s*, M	Axial Ratio [mean]		*R_h_* (nm) [mean]	
**KR**	0.5	3.13	3.25	[3.28]	2.10	2.06	5.64	[5.88]	5.75	[5.70]
	0.25	3.19	3.31		1.85	2.03	6.11		5.65	
**EG**	1	3.11	3.22	[3.25]	1.98	2.08	5.30	[5.63]	5.81	[5.75]
	0.5	3.16	3.28		1.94	2.04	5.96		5.80	
**ER**	0.5	3.22	3.33	[3.37]	1.81	2.01	6.40	[6.64]	5.61	[5.55]
	0.25	3.22	3.34		2.02	2.01	6.40		5.60	
	0.125	3.32	3.44		1.39	1.95	7.14		5.44	
**KG**	1	3.09	3.20	[3.26]	2.04	2.09	5.12	[5.67]	5.84	[5.74]
	0.5	3.13	3.25		2.00	2.06	5.64		5.75	
	0.25	3.20	3.32		1.51	2.02	6.26		5.62	

1 Symbols in this table: *s* = sedimentation coefficient (in Svedbergs); *f/f_o_* = frictional ratio from SEDFIT and from the combination of s and molecular mass (M); *R_h_* = hydrodynamic radius; KR, *etc.* refer to CR1 15–25 (KR), *etc.*

Thus, consistent with NMR and SAXS-derived structural models of CCPs 20–23, the Knops blood group allotypic variations in CCPs 24–25 do not induce structural differences in CR1 15–25 detectable by AUC (or dynamic light scattering). These data, taken together with the SAXS and NMR findings for CR1 20–23, are best explained by an open architecture for CR1 15–25, in which contacts between non-adjacent modules are improbable. On the other hand, the sequences of modules (CCPs 15–19 and CCPs 24–25) flanking the elongated section (CCPs 20–23) cannot extend linearly and rigidly in opposite directions (since that would have resulted in a structure with a much higher axial ratio). It therefore remains conceivable that these flanking sequences could cooperate in binding to a large ligand such as C3b or C4b, even though in previous work no binding between long-homologous repeat D (alone) and C3b/C4b was detected [20]. This possibility, whereby long-homologous repeat D contributes to binding C3b/C4b only after the higher affinity site in long-homologous repeat C had been occupied, was explored through binding assays.

### All four CR1 15–25 variants bind equally well to opsonins C3b and C4b

**Figure 5:**
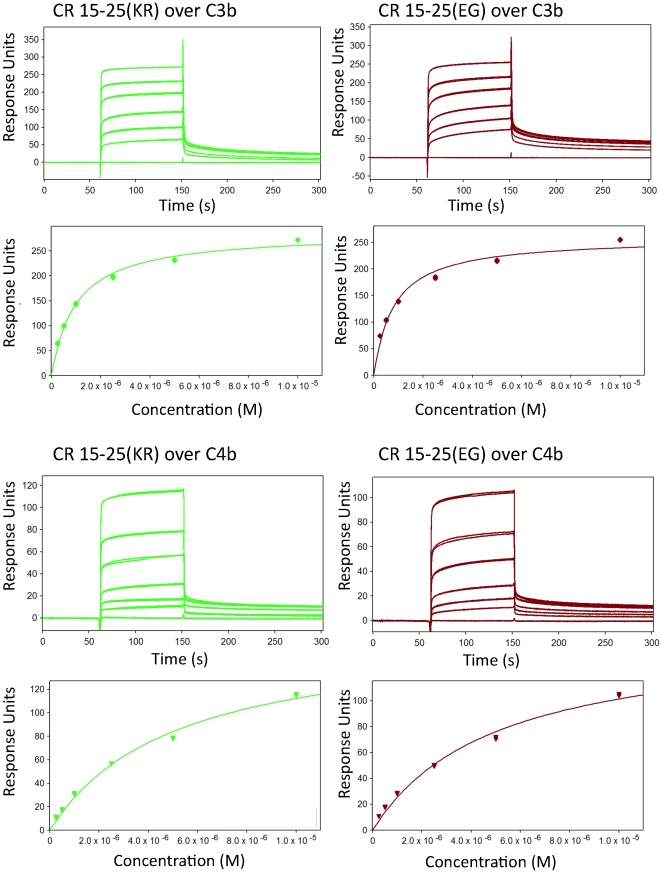
CR1 15–25 variants bind equally well to C3b and C4b. Representative SPR-derived binding curves for the CR1 15–25 variants (as indicated in parentheses) flowed over C3b (upper four panels) or C4b (lower four panels) that were amine-coupled to CM5 chips (see also Figs. S1 and S2). In each case, a set of sensorgrams recorded for a range of CR1 15–25 concentrations (0.25 µM, 0.5 µM, 1 µM, 2.5 µM, 5 µM, 10 µM), are shown above the plot of response versus [CR1 15–25] used to calculate (see Methods) the *K*_D_ value listed in Table 2. CR1 variants that are widespread amongst Caucasoids (KR) or Africans (EG) bind equally both to C3b and C4b.

We investigated whether variant residues in CCPs 24–25 modulate the C3b/C4b-binding ability of CR1 functional site 2. Experiments based on SPR (see Fig. 5, and Figs. S2, S3) provided no evidence for such a proposition. All four CR1 15–25 variants bind similarly well to immobilized C3b, with *K*_D_ values in the range of 0.8–1.1 µM, and to C4b, with *K*_D_ values of 5.0–5.3 µM (Table 2). These data may be compared to *K*_D_ values for CR1 15–17 of 1.1 µM (C3b) and 3.2 µM (C4b), measured on the same C3b- and C4b-loaded flow cells of the CM5 sensor chip (Figs. S2, S3). All the truncations bind less tightly to C3b and C4b than sCR1 (measured on the same CM5 chip, *K*_D_ = 0.53 µM for C3b and *K*_D_ = 0.91 µM for C4b; (Figs. S2, S3)); presumably this reflects the avidity provided by the multiple sites present in the full-length ectodomain. To provide independent validation of these results that were based on studies of binding to covalently immobilized (amine-coupled) C3b and C4b, an ELISA assay was conducted in which C3b or C4b were adsorbed to polystyrene microtitre plates (Fig. S4). These experiments also failed to distinguish between the binding properties of CR1 15–25 variants. We concluded that the variant residues of CCP 25 have neither direct nor indirect roles in engagement of C3b and C4b by site 2.

**Table 2:** Tabulated data derived from SPR experiments.

CR1 fragment	C3b *K*_D_ (µM) [R_max_]^1^	C4b *K*_D_ (µM) [R_max_]	C1q *K*_D_ (µM) [R_max_ ]
**CR1 15–17**	1.06±0.02 [149±1]	3.20±0.07 [94±1]	0.82±0.02 [51.4±0.3]
**CR1 1–3**	38±2 [128±3]	11.2±0.2 [133±1]	30.9±0.9 [94±1]
**CR1 15–25(KR)**	0.99±0.01 [269±1]	5.1±0.1 [169±2]	5.7±0.3 [62±2]
**CR1 15–25(ER)**	1.09±0.01 [291±1]	5.3±0.1 [170±2]	5.7±0.3 [61±2]
**CR1 15–25(KG)**	1.05±0.01 [279±1]	5.2±0.1 [162±2]	5.5±0.3 [54±2]
**CR1 15–25(EG)**	0.80±0.01 [295±1]	5.0±0.1 [152±2]	5.1±0.3 [47±2]
**sCR1**	0.53±0.01 [169±1]	0.91±0.01 [121±1]	8±1 [39±3]

1 R_max_ = fitted maximum response, expressed in response units.

### The CR1 15–25 variants have identical abilities to regulate complement

**Figure 6:**
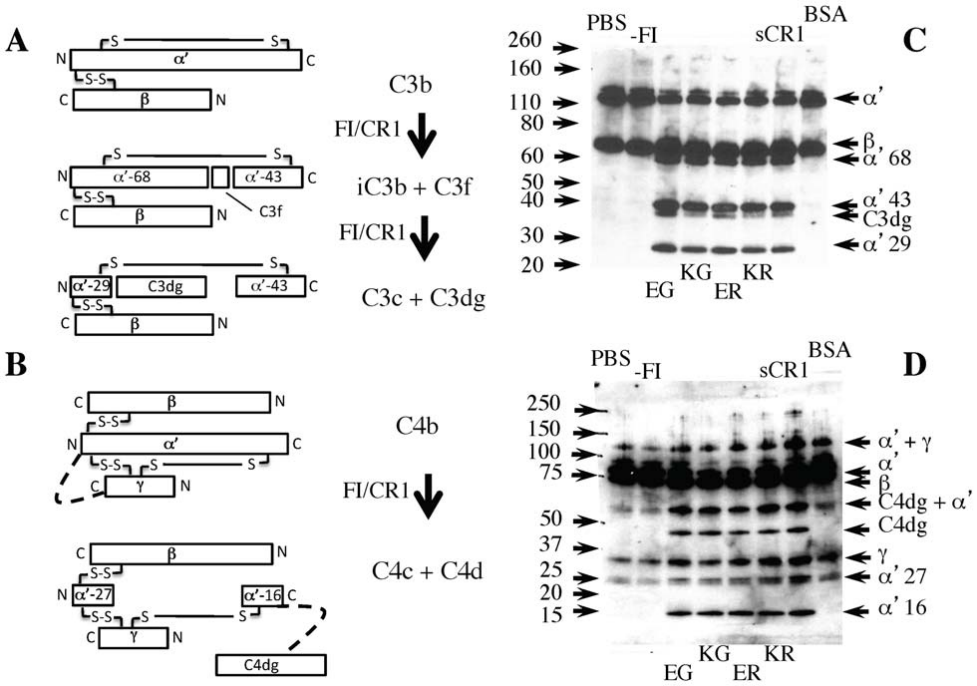
All four CR1 15–25 variants have similar cofactor activity. The products of CR1/Factor I action on (**A**) C3b and (**B**) C4b. In the schematics, disulfides linkages (S-S) are shown as solid lines, and suspected non-disulfide covalent-linkage artifacts (thought to originate from dimerisation in the C4b samples) shown as dotted lines. The gels show the results of SDS-PAGE performed on biotinylated C3b (**C**) or C4b (**D**) (see Methods) following incubation (for one hour) with factor I and the CR1 15–25 variant indicated below (KR, KE *etc.*). A positive control is provided by soluble CR1 (sCR1); negative controls are either bovine serum albumin (BSA) or minus factor I (-FI). Notes: the 2008-Da C3f runs off the gel; the C4b γ chain stains poorly; in the initial C4b sample, degradation products at 25 kDa and 67 kDa are present as contaminants; the reaction was deliberately stopped prior to completion to allow a comparison to be made between the variants. See Figure S3 for ELISA results.

Functional site 2 of CR1 is the predominant locus of cofactor activity for factor I-catalyzed proteolysis of C3b and C4b (see Figs. 6A,B). Cofactor activity very likely involves contacts between CR1 and both C3b (or C4b) and factor I [35]. We showed (above) that the long-homologous repeat C-copy of site 2 engages with C3b and C4b independently of long-homologous repeat D. Nonetheless it remained feasible that Knops blood group variations in CCPs 24/25 influence recruitment, or activation [35], of factor I to CR1:C3b or CR1:C4b complexes. We conducted fluid-phase cofactor assays in which cleavage products of CR1/factor I, acting on C3b or C4b. were detected in a qualitative manner using SDS-PAGE, ensuring reactions had not proceeded to completion. In all CR1 15–25 lanes and the sCR1 lane, the pattern and strengths of bands corresponding to cleavage products (following a 60-minute incubation) appeared to be identical in the case of C3b (Fig. 6C) and, likewise, in the case of C4b (Fig. 6D). Thus all the recombinant variants had co-factor activity for the cleavages made by factor I needed to generate C3dg or C4d. No differences could be detected between the variants in the amounts or nature of product generated.

### The variants have equivalent affinities for C1q

**Figure 7:**
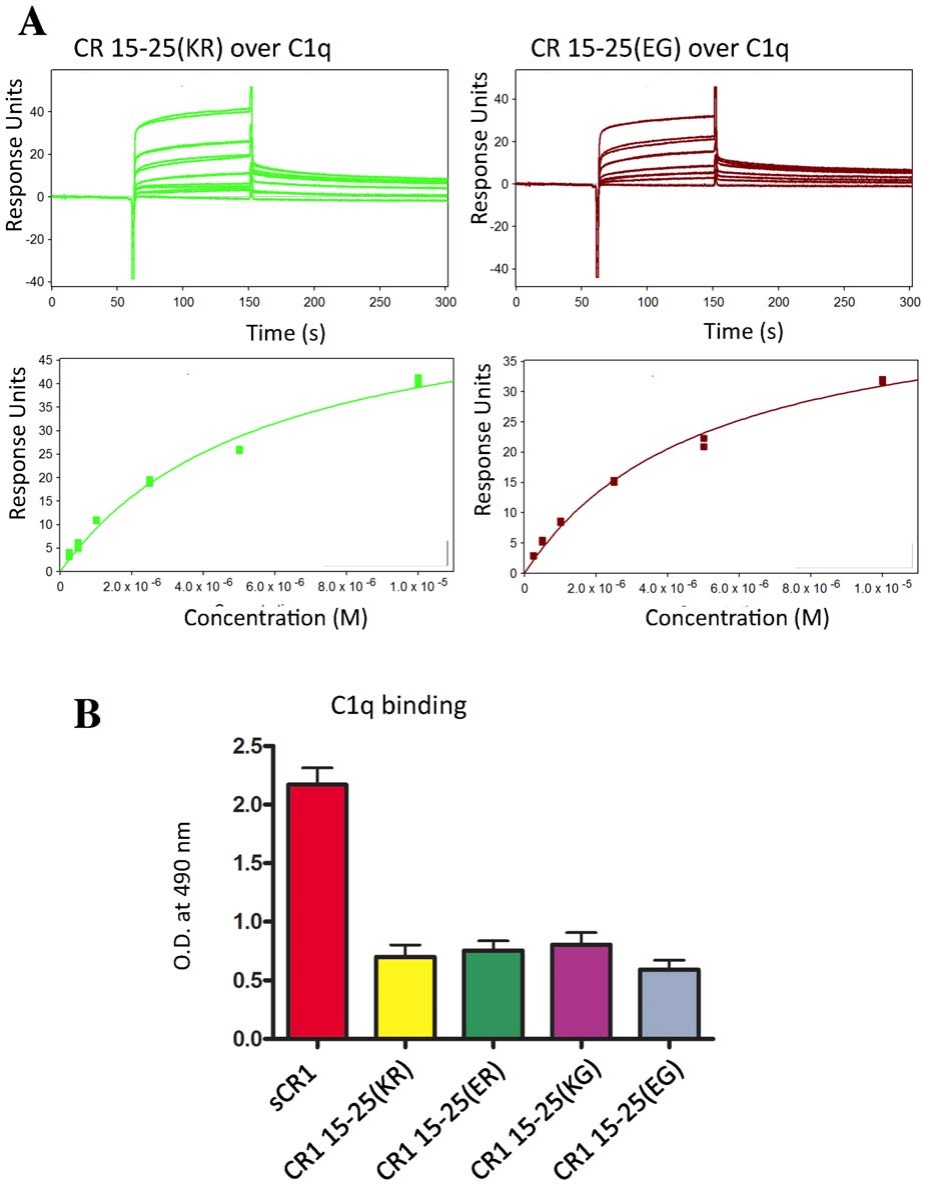
Affinities for C1q. (**A**) Representative SPR-derived binding curves for CR1 15–25 variants flowed over a CM5 chip loaded (via amine coupling) with C1q (see also Fig. S4). *Upper:* sensorgrams recorded for a range of CR1 15–25 concentrations (as in Fig. 3). *Lower:* plots of response versus [CR1 15–25] used to calculate (see Methods) the *K*_D_ values listed in Table 2. CR1 variants that are widespread amongst Caucasoids (KR) or Africans (EG) bind equally to C1q. (**B**) Data obtained by ELISA showing that C1q (140 ng.mL^−1^, average of three experiments) bound equally to each of the four variants of CR1 15–25 (adhered to the micro-titer plate). In these experiments, sCR1 was used as a positive control. Error bars represent standard errors of the mean.

Interaction of CR1 with C1q was reported previously, along with suggestions that long-homologous repeat D (CCPs 22–28) contributes to this binding event cases [32], [36], [37]. We measured affinities of the four CR1 15–25 variants for C1q that had been immobilized, via amine coupling, on the CM5 sensor chip surface (under the same conditions and in the same series of experiments used to obtain *K*_D_ values for C3b and C4b) (Fig. 7A, Table 2). Measured values lay in a narrow range of 5–6 µM, comparable with a value of 8 µM for sCR1 (Fig. S5). CR1 1–3 (site 1) bound weakly (*K*_D_>30 µM (not shown) but in this assay CR1 15–17 (site 2) bound better than sCR1 (*K*_D_<1 µM) while the nine-CCP construct CR1 17–25 (*i.e.* missing CCPs 15 and 16) did not bind at all (Fig. S5); these data suggesting that C1q binds to site 2 require further verification. In any case, the results of SPR strongly indicate that the *McC* and *Sl*-encoded variations had small or negligible effects on binding of C1q. Very similar results were obtained using an ELISA (see Fig. 7B) in which soluble C1q binding to CR1 truncations adsorbed on a micro-titer plate was measured.

### PfRh4 invasion pathway

**Figure 8:**
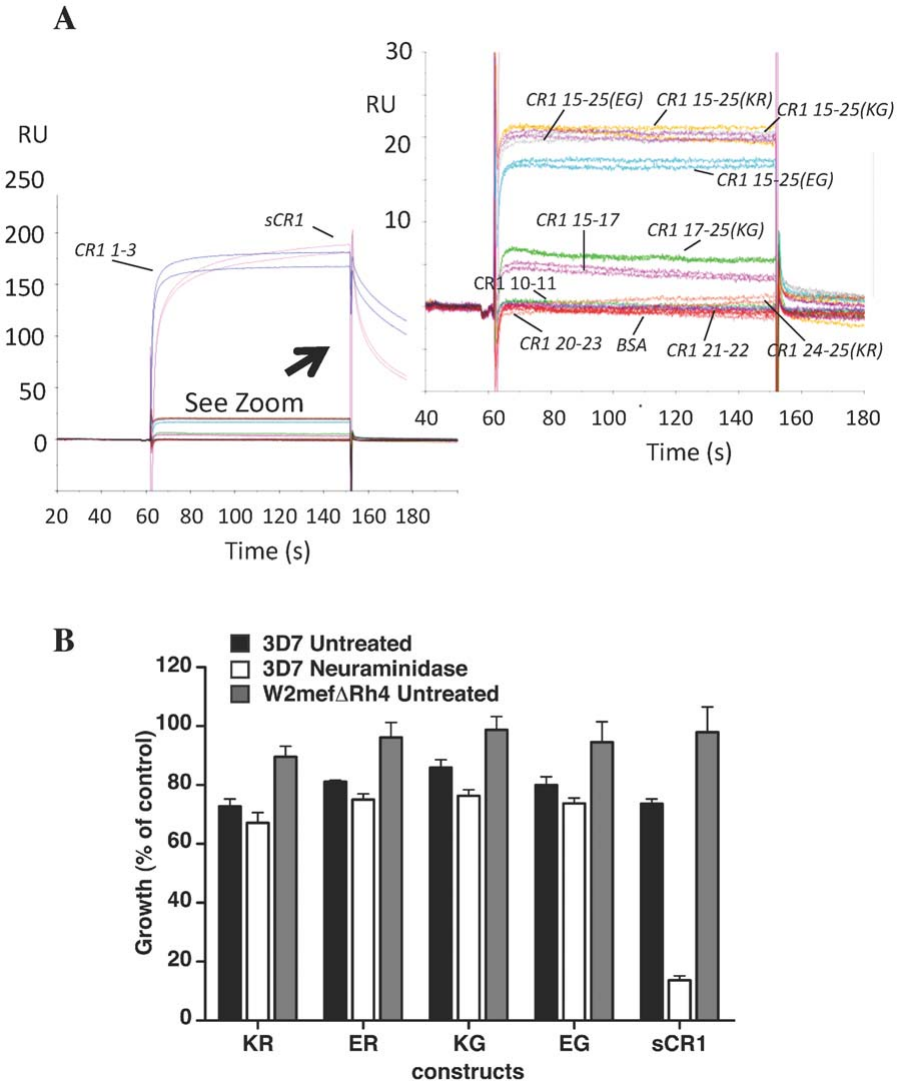
None of CR1 15–25 variants interact with *P. falciparum* protein Rh4. (**A**) In an SPR experiment, no CR1 15–25 variant had significant affinity for immobilized Rh4.9, unlike positive controls sCR1 and CR1 1–3 (26). Other constructs lacking CCPs 1–3 (CR1 10–11, CR1 15–17, CR1 20–23, CR1 21–22 and CR1 24–25(KR)) were likewise lacking in affinity for Rh4.9 and, like BSA, served as negative controls. (**B**) The PfRh4 invasion pathway was not inhibited in the presence of CR1 15–25 variants. Parasite strains 3D7 (black bars for untreated, white bars for *nm*-treated erythrocytes) and W2mefΔRh4 (grey bars, untreated erythrocytes) were tested in growth assays in the presence of final concentrations of 0.5 mg/ml of the four CR1 15–25 variants (KR, ER *etc.*). Growth (% of control) on the y-axis refers to the % parasitemia in the presence of CR1 constructs relative to the % parasitemia with the addition of PBS (arbitrarily set to be 100%).

Erythroid CR1 is the receptor required for the major sialic acid-independent route for invasion by multiple strains of *P. falciparum* [9], [10], [11], [26]. The key *P. falciparum* protein in this invasion pathway, PfRh4, was previously shown to bind to CR1 CCPs 1–3 (site 1), but not to the highly similar CCPs 15–17 (site 2) nor to the Caucasoid variant (Lys^1590^/Arg^1601^) of CR1 15–25 that includes CCPs 22–24; these modules are similar in sequence to both CCPs 1–3 and CCPs 15–17 [26]. We examined the previously unexplored possibility that PfRh4 engages with other, non-Caucasoid, variants of CR1 15–25. According to SPR, none of the CR1 15–25 variants (like CR1 15–17, CR1 24–25(KR) and other negative controls) exhibited more than residual affinity for an immobilized recombinant version of PfRh4 (PfRh4.9) corresponding to its erythrocyte-binding N-terminal domain [38], unlike the positive controls, CR1 1–3 and sCR1 (Fig. 8A). To investigate whether the non-Caucasoid CR1 15–25 variants nevertheless interfered with the PfRh4-CR1 invasion pathway, their effects on parasite growth were monitored. As was observed previously, invasion of both untreated and neuraminidase-treated erythrocytes by 3D7 strain was inhibited by addition of sCR1, whereas invasion of untreated erythrocytes by the parasite strain W2mefΔRh4, which lacks a functional CR1-PfRh4 pathway, was unaffected by the presence of sCR1 (Fig. 8B) [26]. These results served as a control for experiments with the CR1 15–25 variants [9], [11] (see Fig. 8B). In agreement with the SPR study, none of the CR1 15–25 variants (unlike the positive control, sCR1) inhibited invasion of *nm*-treated erythrocytes by *P. falciparum*. Thus the Knops blood group antigens on CCPs 24/25 had no influence on the susceptibility of erythrocytes to invasion by *P. falciparum*, consistent with a location within the CR1 molecule that is remote both in the sequence and in space from the key PfRh4-binding site in CCPs 1–3 (site 1) [26].

### All variants are equally implicated in *P. falciparum* rosetting

**Figure 9:**
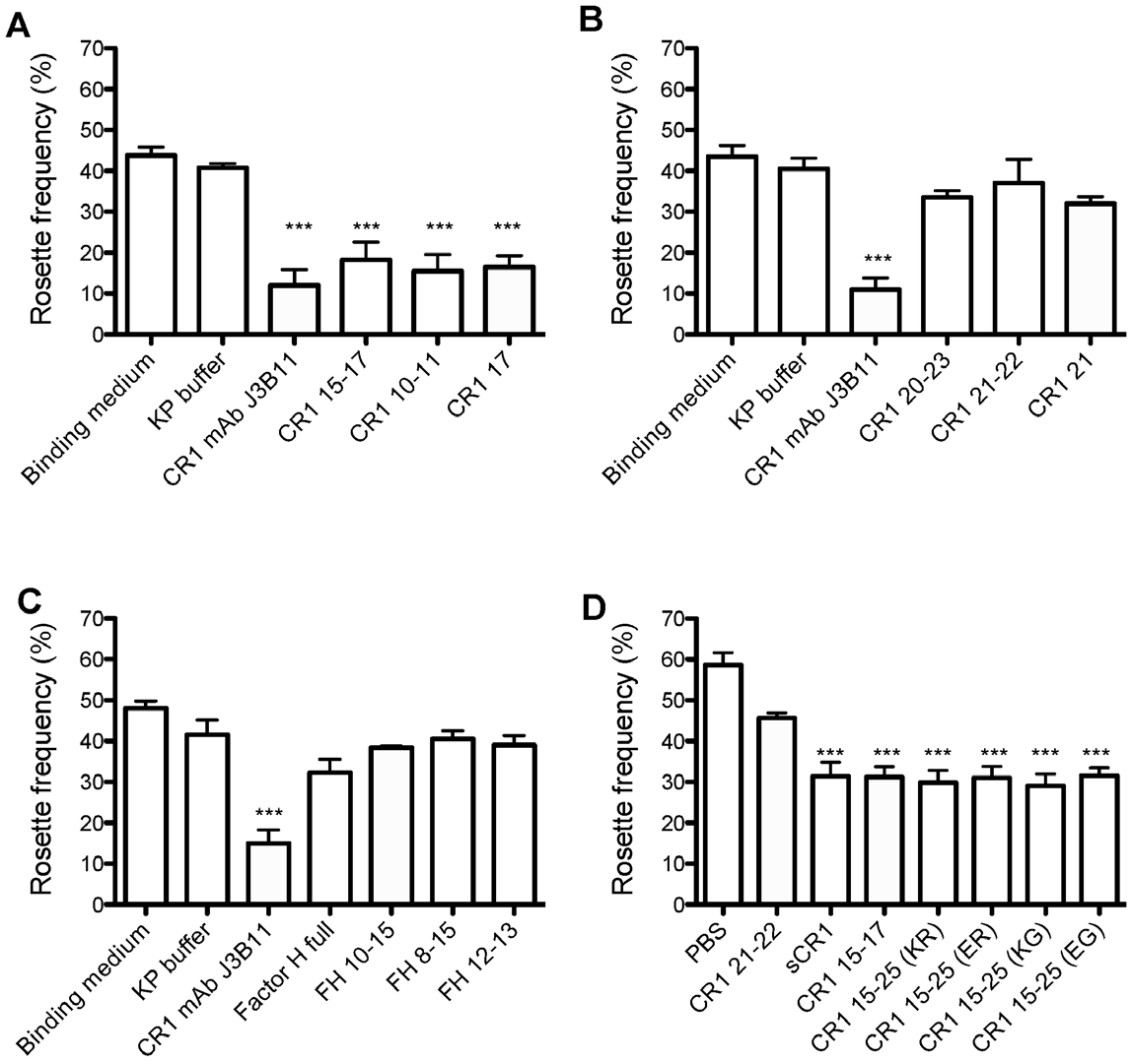
CR1 15–25 variants do not differ in ability to disrupt *P. falciparum* rosetting. (**A**) Both CR1 10–11 (identical to CR1 17–18) and single-module CR1 17 can disrupt rosetting of *P. falciparum* clone IT/R29, as can anti-CR1 antibody (J3B11) and CR1 15–17 [27] used here as positive controls. The truncation mutants corresponding to CCPs 20–23 (**B**) and the CR1 homologue, factor H (FH, full-length or fragments as indicated below) (**C**) did not disrupt rosetting, and served as negative controls. (**D**) A comparison of the four CR1 15–25 variants suggested they were all equally as effective as full-length sCR1 and CR1 15–17 in terms of rosette disruption. KP: 50 mM potassium phosphate buffer; The mean and standard error of at least four experiments for each graph are shown. *** p<0.001 by ANOVA and Tukey's multiple comparison test.

Previous work implicated site 2 (CCPs 8–10 or CCPs 15–17) in the rosette-mediating interaction between erythroid CR1 and PfEMP1 expressed on the surface of infected erythrocytes [27]. We were unable (data not shown) to detect binding between any of the CR1 truncations (including sCR1) and immobilized recombinant versions of the N-terminal duffy binding-like domain from PfEMP1 [39] (in the same set of SPR experiments used to measure the affinity of sCR1 for Rh4.9). Nonetheless, we were able to elaborate on previous findings by confirming that not only was rosetting inhibited by sCR1 and by CR1 15–25, but it was also ameliorated by CR1 15–17 (as shown previously) and even by CR1 17 alone (Fig. 9A). This appeared to be a module-specific effect since rosetting was not inhibited by CR1 21, CR1 21–22 or CR1 20–23 (Fig. 9B), nor by full-length factor H or several factor H fragments (Fig. 9C). Importantly, we could not detect any difference between the four variants of CR1 15–25 in terms of their ability to disrupt rosetting (Fig. 9B).

## Discussion

Complement receptor-type 1, a rare example of a protein whose ectodomain is composed entirely from modules of the same type, is a product of gene duplication and exon shuffling [40]. This evolutionary heritage is particularly apparent amongst CR1 size-variants (190–280 kDa), which possess different numbers of homologous blocks of seven CCPs arranged in long-homologous repeats [19]. In the 30-CCP ectodomain of the most common (gene frequency of ∼0.85) size-variant, four tandem N-terminal long-homologous repeats (A–D), are followed by two membrane-proximal CCPs. Each CCP contains about 60 amino acid residues organized in a β-strand-rich compact unit stabilized by pairs of disulfides between invariant cysteines (Cys I-Cys III, Cys II-Cys IV) [41], [42]. We investigated structural and functional consequences of Knops blood group antigenic variations of CR1 likely to be under geographical region-specific selective pressure resulting in non-uniform distribution of allotypes among global populations. These involve loss or gain of charge through substitution of residues predicted to be surface-exposed (see Fig. 1) and thus the E1590/G1601 (*McC^b^/Sl2*, common in sub-Saharan African) and K1590/R1601 (*McC^a^/Sl1*, predominant in Caucasoids) [2] variants will differ significantly in the electrostatic surfaces presented by their CCPs 24–25 regions (in long-homologous repeat D, Fig. 1) [43].

Linkers of just four amino acid residues join long-homologous repeats A and B, and long-homologous repeats B and C, but an eight-residue linker connects the last residue of long-homologous repeat C (*i.e.* consensus Cys IV of CCP 21) to the first of long-homologous repeat D (*i.e.* consensus Cys I of CCP 22). We looked for, but found no evidence of, structural or functional interactions between long-homologous repeats D and C despite the potential for this double-length linker between long-homologous repeats to allow or promote a close-to-180-degree bend in the molecule. Indeed, our results suggest the modules flanking this linker adopt an extended arrangement consistent with a stalk-like or spacer role for long-homologous repeat D, helping to project the functional sites-bearing CCPs 1–21 away from the membrane and clear of the glycocalyx of erythrocytes, where over 85% of human CR1 resides [2]. Thus, we infer that single or double-residue substitutions in long-homologous repeat D of surface-exposed residues are unlikely to have structural or electrostatic consequences for long-homologous repeats A–C.

Our SPR and ELISA-derived binding data are fully consistent with a large body of previous work suggesting N-terminal modules of long-homologous repeats A–C cooperate in interacting with the activated products of C3 and C4 (C3b and C4b) and their complexes, while long-homologous repeat D (the location of variant residues 1590 and 1601) does not engage directly with these ligands [17], [20], [21], [23], [24]. Long-homologous repeats A, B and C each carry an N-terminal set of three modules that presumably bind to surface-tethered C3b/C4b in a manner similar to one another and to factor H-modules 1–3 (*i.e.* the long axes of modules aligned with the long axis of C3b and with the N-terminal of the three modules distal to the surface) [44]. Thus it is easy to envisage long-homologous repeats A–C, borne on the long-homologous repeat D stalk, adopting an architecture that has a quasi-three-fold axis of symmetry. Our results indicated that CCPs 15–25 encompass binding sites for C1q, which is multi-subunit activator of the classical pathway. Our studies suggested CCPs 15–17 make a major contribution to C1q binding whereas previous studies ascribed more importance to long-homologous repeat-D [32], [36], [37]; in our studies CCPs 26–28 of long-homologous repeat D were absent so we did not make a direct comparison between these two possibilities.

Consistently with our structural findings, we demonstrated that *Sl* and *McC*-encoded sequence variations within long-homologous repeat-D appear to have no effect on a range of CR1 properties resident in long-homologous repeats A–C including: C3b-binding and C4b-binding, vital for the long-recognized key biological role of human CR1 as the immune adherence receptor [45]; cofactor activity for factor I, needed for protection of erythrocyte surfaces from C3b proliferation and its potentially cytolytic consequences [46]; interactions with *P. falciparum*-derived erythrocyte-borne PfEMP1, required for rosetting [12], [27]; and engagement by the *P. falciparum* adhesin PfRh4 for *P. falciparum* invasion of erythrocytes [11]. Our results additionally demonstrated that binding to CR1 of C1q occurs entirely independently of the *Sl* and *McC*-encoded variations.

For rosette-disruption assays we chose parasite clone R29, on the grounds that it is the best-characterized CR1-sensitive *P. falciparum* rosetting strain [12], [27]. In previous work, no differences were observed between strains in response to various agents such as the CR1 mAb J3B11 and recombinant CR1 15–17 [27]; on this basis it was decided not to initiate extensive testing of other strains for rosette disruption. Although our experiments were not conducted on variant versions of the full-length ectodomain, this is unlikely to have eroded their relevance in respect of the assays performed in this study. Interactions between long-homologous repeat D and long-homologous repeats A or B, but not C, would require an unfeasibly contorted architecture for the CR1 molecule. It also seems unlikely that CCPs 26–30 (absent in our truncation) are a requirement for a putative interaction between long-homologous repeats D and C. On the other hand, our focus on soluble truncations as opposed to membrane-bound variants does leave open a number of untested possibilities. In particular, although we observed no differences in self-associative properties of CR1 15–25, it is possible that a stalk-like long-homologous repeat D is important for mediating the clustering of CR1 molecules [47]. The relevance of CR1 clustering to merozoite invasion or rosetting has not been investigated but it has been reported to be important for cell deformability, motility of cells in microvasculature and hence the immune clearance role of CR1. It is also possible that *Sl* and *McC*-encoded sequence variations affect a putative interaction, of unknown physiological significance, with mannan binding lectin [37]. Moreover, CR1 is displayed on other cell-types than erythrocytes and may have other functions than those tested in this study. Finally, we used C4b from pooled plasma and therefore we did not take account of polymorphic variation in C4 that also exhibits a non-uniform distribuion across geographical regions of origin.

African-derived populations are characterized by a slightly higher (0.12 versus 0.18) incidence of a larger size-variant of CR1 [1], and high copy numbers of CR1 on erythrocytes [48] as well as increased frequency of the *Sl2* and *McC^b^* alleles [3]. The current study makes a correlation between the last of these sources of polymorphic variation and malaria seem less likely. Nor does the data support hypotheses based on differential abilities amongst these Knops blood group antigenic variants to protect erythrocytes against cytolytic complement attack. Our C3b- and C4b-binding data suggest that all the variants will be equally good at clearing immune complexes, although our studies of soluble fragments did not take into account the possible roles in this respect of CR1 clustering. Overall, a likely scenario is that the source of selective pressure on these variants in long-homologous repeat D arises from less-well understood interactions between CR1 and pathogenic organisms. For example, elevated frequencies of *McC^b^* and *Sl2* alleles have been correlated to resistance to *Mycobacterium* tuberculosis infections [49].

## Materials and Methods

### Protein production

Recombinant versions of truncated CR1 (the Caucasian, *i.e.* Lys1590, Arg1601, variant) were produced in a *Pichia pastoris* expression host using methods described previously. In brief, DNA coding for CCPs 21 (E1317-R1392), CCPs 21–22 (E1317-S1456), CCPs 20–23 (S1257-I1516) or CCPs 15–25 (T940-S1647) (numbering refers to unprocessed initial gene product) was PCR-amplified from CR1 cDNA then ligated into the TOPO plasmid vector (Invitrogen) that was used to transform Top10 *E. coli* cells (Invitrogen). Amplified and subsequently extracted (using the QIAprep Miniprep Kit, Qiagen) DNA was digested with *Pst*I and *Xba*I and the insert was ligated into the pPICZ αB vector (Invitrogen) downstream of the DNA for the *Saccharomyces cerevisiae* α-mating factor secretion signal. Plasmids were amplified in *E. coli* cells, linearised and used to transform *P. pastoris* KM71H cells for production of protein that is directed into the secretory pathway. Purification from media after cell removal was achieved by cation-exchange chromatography followed by a second ion-exchange chromatography step and gel-filtration chromatography. Proteins exhibiting N-linked glycosylation were treated after the first purification step with the endoglycosidase Endo H_f_ (New England Biolabs). Purification was monitored by sodium dodecyl sulfate-polyacrylamide gel electrophoresis (SDS-PAGE) performed under both reducing and non-reducing conditions. All of the recombinant truncations of CR1 carried the expression artifact EAEAAG at their N-termini. For isotopic enrichment, cells were grown in minimal media supplemented with ^15^N-ammonium sulfate and (for double labeling) ^13^C-glucose (during growth phase) and ^13^C-methanol (during induction phase). A ^15^N,^2^H-labelled sample of CR1 20–23 was produced by supplementing buffered minimal glycerol with ^15^N ammonium sulfate and also replacing H_2_O with 98% v/v D_2_O.

Starting with DNA for CR1 15–25(KR), the QuikChange Kit (Stratagene) along with the appropriate oligonucleotides was used to introduce the nucleotide substitutions needed to encode the Knops blood group antigenic variations (*i.e.* single substitutions K1590E and R1601G and double substitution K1590E/R1601G).

### NMR spectroscopy

All NMR data were collected in 5-mm NMR tubes on an Avance800 NMR spectrometer (Bruker) fitted with a cryoprobe, processed using TOPSPIN (Bruker), then analyzed by utilizing the ANALYSIS module of the common computing protocol for NMR (CCPNMR) software package [50]. Two-dimensional ^15^N-^1^H HSQC spectra and three-dimensional triple-resonance spectra (CACBNH and CACB(CO)NH) [51] were acquired at 37°C on a 300 µM sample of the ^13^C,^15^N-labelled CR1 21–22 protein in 20 mM deuterated sodium acetate, pH 4.5, containing 10% v/v D_2_O and 0.02% w/v NaN_3_. Two-dimensional ^15^N-^1^H HSQC and TROSY spectra were collected on 233 µM ^15^N-labelled CR1 21 and 2 mM ^15^N-labelled CR1 21–22 samples, respectively, under the same conditions. Partial backbone and ^13^Cβ resonance assignments were determined for CR1 21–22 according to standard procedures.

### Analytical Ultracentrifugation

Sedimentation velocity analysis (at 18±0.5°C) was performed on 0.4-ml protein samples (0.2–0.4 mg.ml^−1^ in PBS, pH 7.5), on a XL-A analytical ultracentrifuge (Beckman). A series of radial scans of optical density (279 nm) across the centrifuge cell was collected (Proteome Lab software, Beckman-Coulter). An initial scan was performed immediately upon attaining the set rotor speed (45,000 rpm); 80 subsequent scans were recorded at two-minute intervals. Each resulting data set was analyzed (SEDFIT [52]) yielding values of the *s*-distribution parameter *c*(*s*) as a function of sedimentation coefficient (*s*). For non-linear fitting of optical density values *versus* radial distance in SEDFIT, the resolution was set to 150, and a confidence factor set to 0.68. An average value for the frictional ratio (*F*) was determined over a series of fits and used as a default for all fittings; baseline, meniscus, and cell base radial positions were floated. The final profile, stored as a continuous distribution, was analyzed within the general software set for data analysis, “pro Fit” (QuantumSoft, Zurich). The maximal value of the *c*(*s*) function was used to define the sedimentation coefficient of the target molecule. A value of the partial specific volume (0.721±0.001 ml.g^−1^ for all four allotypic variants) was computed (using SEDNTERP [53]), which was also employed to compute density and viscosity properties of the buffer solution, enabling correction of raw *s* values to standard conditions of solvent viscosity and density (*s*_20,w_). The diffusion coefficient (an inverse function of *F*) of each species was estimated in the conventional manner from s, M and partial specific volume within a locally written utility for hydrodynamic calculations, Biomols 2. From each of the *F* values determined it was straightforward to compute (using SEDNTERP), on the basis of an assumed ‘typical’ (for protein) value of solvation (1.4, vol/vol), an estimate for the overall ‘shape’ of the solute particle, modeled as a prolate ellipsoid of revolution.

### Small-angle X-ray scattering (SAXS)

Synchrotron radiation X-ray scattering data were collected on the ×33 beam line of the EMBL (DESY, Hamburg) [54], [55], using a MAR345 image plate detector (MarResearch, Norderstedt, Germany) and 120-s exposure times. Solutions of CR1 20–23 (1.9 and 3.3 mg.ml^−1^) were measured (10°C) in 50 mM potassium phosphate (KP) buffer, pH 7.4. The sample-to-detector distance (2.7 m) covered a range of momentum transfer 0.1<*s*<6.0 nm^−1^. No radiation damage was observed during a second 120-s exposure. Data from the detector were normalized to the transmitted beam intensity, averaged and the scattering of buffer solutions subtracted. Difference curves were scaled for solute concentration. Data manipulations were performed using *PRIMUS* [56]. Forward scattering *I*(0) and radius of gyration (*R_g_*) were determined from Guinier analysis [57], assuming that at very small angles (*s*<1.3/*R_g_*), intensity, *I*(*s*),  = *I*(0)exp(-(*sR_g_*)^2^/3). These parameters were also estimated from the full scattering curves using the indirect Fourier transform method implemented in GNOM [58], along with the distance-distribution function *p(r)* and maximum particle dimensions *D_max_*. Molecular weight was estimated by comparing extrapolated forward scattering with that of a reference solution of bovine serum albumin. Due to uncertainty in MWt estimation resulting from uncertainty in measured protein concentrations, an excluded volume of solutes was determined from the *ab initio* modeling program DAMMIF [59]. For globular proteins, this hydrated particle volume in nm^3^ is approximately 1.5 to 2 times the MWt in kDa. Low-resolution shape envelopes for CR1 constructs were determined by *ab initio* bead-modeling in DAMMIF [59]. The results of multiple DAMMIF reconstructions were aligned using SUPCOMB [60] to determine the most representative model from each of the *ab initio* methods. Averaged DAMMIF models were also determined using DAMAVER [56] and then adjusted, so that they agree with the experimentally determined excluded volume, using DAMFILT [61].

### Surface plasmon resonance (SPR)

Duplicate SPR experiments were carried out (25°C) on a Biacore T100 (GE Healthcare). C3b, C4b and C1q (Complement Technology, Texas) were immobilized using standard amine coupling, and dissociation constants measured as described [62]. Briefly, 1620 response units (RUs), 1518 RUs and 2125 RUs of C3b, C4b and C1q, respectively, were immobilized on three of four flow cells of a Biacore Series S carboxymethylated dextran (CM5) sensor chip. Experiments were performed by flowing over the chip 30 µl.minute^−1^ analyte solution containing a range of concentrations of CR1 fragments that had been dialyzed into 10 mM Hepes-buffered 150 mM saline with 3 mM EDTA and 0.05% v/v polysorbate 20 (HBS-EP; pH 7.4). Regeneration was achieved by flowing 1.0 M NaCl over chip surfaces. Dissociation constants (*K*_D_s) were calculated (using Biacore T100 evaluation software v. 2.0) by fitting steady-state binding levels derived from background-subtracted traces to a one-to-one stoichiometry steady-state model. For blank subtractions, a reference surface was prepared by performing two consecutive dummy amine-coupling reactions in the absence of any proteins.

### Parasite culture and growth inhibition assays

*P. falciparum* asexual stages were maintained in human O+ erythrocytes in RPMI-HEPES medium with 50 µg.ml^−1^ hypoxanthine, 25 mM NaHCO_3_, 20 µg.ml^−1^ gentamicin, and 0.5% w/v Albumax II (Gibco; Invitrogen) in 1% O_2_, 4% CO, and 95% N_2_ at 37°C and synchronized by standard methods. 3D7 is a cloned line derived from NF54 obtained from David Walliker, Edinburgh University. W2mef is a cloned line derived from Indochina III/CDC strain. W2mefΔRh4 was derived from W2mef by genetic disruption of the *PfRh4* gene as described [63] Growth inhibition assays (GIA) were performed as described, with the following modifications [64]. Neuraminidase (66.7 mU/ml)-treated or normal erythrocytes in culture medium were inoculated with late-trophozoite stage parasites to give a parasitemia of 1% and hematocrit of 1% in a volume of 50 µl. CR1 fragments or PBS were added at the beginning of the assay. Growth assays were performed in 96-well round-bottom microtiter plates (Becton Dickinson) over one cycle of parasite growth. After 48 hours, the parasitemia was determined by flow cytometry of ethidium bromide-stained trophozoite-stage parasites using a FACSCalibur (BD Biosciences) and a plate reader. For each well 40,000 cells or more were counted. Growth was expressed as mean parasitemia obtained from triplicate readings. At least two to four independent assays were performed, each in triplicate. Growth (% of control) refers to the % parasitemia in the presence of CR1 constructs relative to the % parasitemia with the addition of PBS (arbitrarily set to be 100%).

### Rosetting assays

*P. falciparum* parasites (clone IT/R29) were cultured and selected for rosetting as described previously [39], [65]. The red cells in which the parasites are grown for the rosetting experiments were all group O and different donors were used for different replicate experiments. The red cells were used for parasite culture up to 10 days after drawing. Our unpublished data show that donor red cells form rosettes well up to 10 days, but rosetting starts to reduce after that time. For rosette-disruption assays, parasite cultures were pre-stained with 25 µg.ml^−1^ ethidium bromide then washed once and re-suspended at 2% hematocrit in bicarbonate-free RPMI (Sigma) containing 25 mM Hepes and 10% v/v normal human serum. Aliquots (25–50 µl) of pre-stained culture suspension in micro-centrifuge tubes were mixed with soluble recombinant CR1 proteins in KP buffer to give a final concentration of 20 µM. Mixtures were incubated at 37°C for 30 minutes. During this incubation period, cells were re-suspended at ten-minute intervals by agitation. Subsequently a drop (∼10 µl) of the pre-stained culture suspension was placed on a slide on a slide underneath a 22×22-mm cover slip and viewed with a fluorescence microscope. Infected erythrocytes (that fluoresce orange due to parasite DNA) could be discriminated from non-infected cells. A total of 200 infected red blood cells were counted and scored as either being in a rosette, or not, where a rosette is defined as “an infected cell with two or more uninfected red cells sticking to it”. Only mature (that is, pigmented trophozoite or schizont) infected cells were counted. This allowed calculation of “rosette frequency” as the number of infected cells in rosettes expressed as a percentage of the total number of infected cells counted. The rosette frequency in the presence of recombinant CR1 fragments was compared to that of negative controls with no added protein (an equivalent volume of PBS or RPMI binding medium or KP buffer was added instead of protein). Positive controls showed the rosette disruption obtained by 0.1 µg.ml^−1^ of a monoclonal antibody to CR1 site 2 (J3B11) or 20 µM CR1 15–17 [27]. Rosette frequencies were compared by ANOVA using Graphpad Prism software (La Jolla, USA).

### Complement assays

To estimate ability of CCP 15–25 variants to act as cofactors for C3b cleavage by factor I, 7.5 µl biotinylated C3b (3.3 µg.ml^−1^, *i.e.* 25 pg), 2.5 µl factor I (from Complement Technology) (1.6 µg.ml^−1^, *i.e.* 4 pg) and 10 µl CCP 15–25 (1 µg.ml^−1^ i.*e.* 10 pg), or 10 µl sCR1 (a gift from Henry Marsh, Celdex Pharmaceuticals) (2.7 µg.ml^−1^ *i.e.* 27 pg), were mixed in a final reaction volume of 30 µl of 25 mM phosphate buffer, pH 7.3, containing 50 mM NaCl. For C4b cleavage assays, 7.5 µl biotinylated C4b (5 µg.ml^−1^, 37.5 pg), 2.5 µl factor I (1.6 µg.ml^−1^, 4 pg) and 10 µl CCP 15–25 5 µg.ml^−1^, 50 pg) or 10 µl sCR1 (13.5 µg.ml^−1^, 135 pg), were incubated in a final volume of 30 µl of 25 mM phosphate buffer, with 50 mM NaCl. Incubations (one hour, 37°C) were initiated by addition of factor I and stopped by addition of 10 ml of 4× NuPAGE (Invitrogen) reducing loading buffer followed by heating (95°C) for four minutes. Then 18-µl aliquots were loaded onto 4–12% w/v acrylamide NuPAGE gels (one for C3b cleavages, another for C4b cleavages). Protein bands were transferred to nitrocellulose in a Bio-Rad semi-dry apparatus, blocked overnight in 5% w/v dried milk powder (Lab Scientific) and stained with avidin-horse radish peroxidase (Pierce). Amersham ECL+ reagent was used to develop the blot. C3b and C4b were purchased from Complement Technology and biotinylated using Sulfo-NHS-LC biotin (Pierce). The bands representing cleavage products of the C3b or C4b α' chains were compared by eye.

### ELISAs

For these experiments, C3b and C4b were purchased from Complement Technology (Tyler, Texas). To C3b-coated or C4b-coated micro-titer plates, solutions of 1 mg.ml^−1^ sCR1 or CR1 15–25 variants (in 25 mM NaCl) were added. The primary antibody used for detection was rabbit anti-CR1, and the secondary antibody was a goat anti-rabbit IgG conjugated with horseradish peroxidase. Results of three separate experiments were averaged. For comparisons of C1q binding, sCR1 or CR1 15–25 variants were coated on micro-titer plates. Solutions of C1q (140 ng.ml^−1^ in 75 mM NaCl) (results of three experiments averaged) were added. The primary antibody was rabbit anti-C1q and secondary antibodiy anti-rabbit IgG conjugated with HRP.

## Supporting Information

Figure S1 **Particle size distribution according to dynamic light scattering.** Shown are overlays of dynamic light scattering-derived particle size profiles for the indicated recombinant CR1 fragments (see key). Data were collected using a Zetasizer Nano S system (Malvern Instruments Ltd., UK) on samples of ∼3.5 mg.ml^−1^ protein in phosphate-buffered saline at 25°C.
(TIFF)

Figure S2 **Binding of CR1 constructs to C3b by SPR.** Sensorgrams (*left*) (for a concentration series, see Methods in main text) and response (response units (RU)) versus concentration (M) plots (*right*) for, from *top to bottom*, CR1 15–17 (site 2), CR1 1–3 (site 1), CR1 15–25 (ER), CR1 15–25 (KG) and sCR1. The fitted (see Methods in main text) *K*_D_ values (shown in µM ± error, where the error applies to last significant figure shown, *e.g.* for CR1 15–17, *K*_D_ = 1.06±0.01 µM) are displayed in the plots but also summarized in Table 2, main text.
(TIFF)

Figure S3 **Binding of CR1 constructs to C4b by SPR.** As for Figure S2 except C4b replaced C3b.
(TIFF)

Figure S4 **Binding of CR1 constructs to C3b and C4b by ELISA.** There are no significant differences between the CR1 15–25 variants in terms of their ability to bind to C3b and C4b according to an ELISA (see Methods in main text).
(TIFF)

Figure S5 **Binding of CR1 constructs to C1q by SPR.** As for Figure S2 except C1q replaced C3b.
(TIFF)
