# Supplementary figures and images for: Lack of Evidence from Studies of Soluble Protein Fragments that Knops Blood Group Polymorphisms in Complement Receptor-Type 1 Are Driven by Malaria
Source: PLoS One. 2012 Apr 10;7(4):e34820. doi: 10.1371/journal.pone.0034820 (PMC3323580; doi:10.1371/journal.pone.0034820)

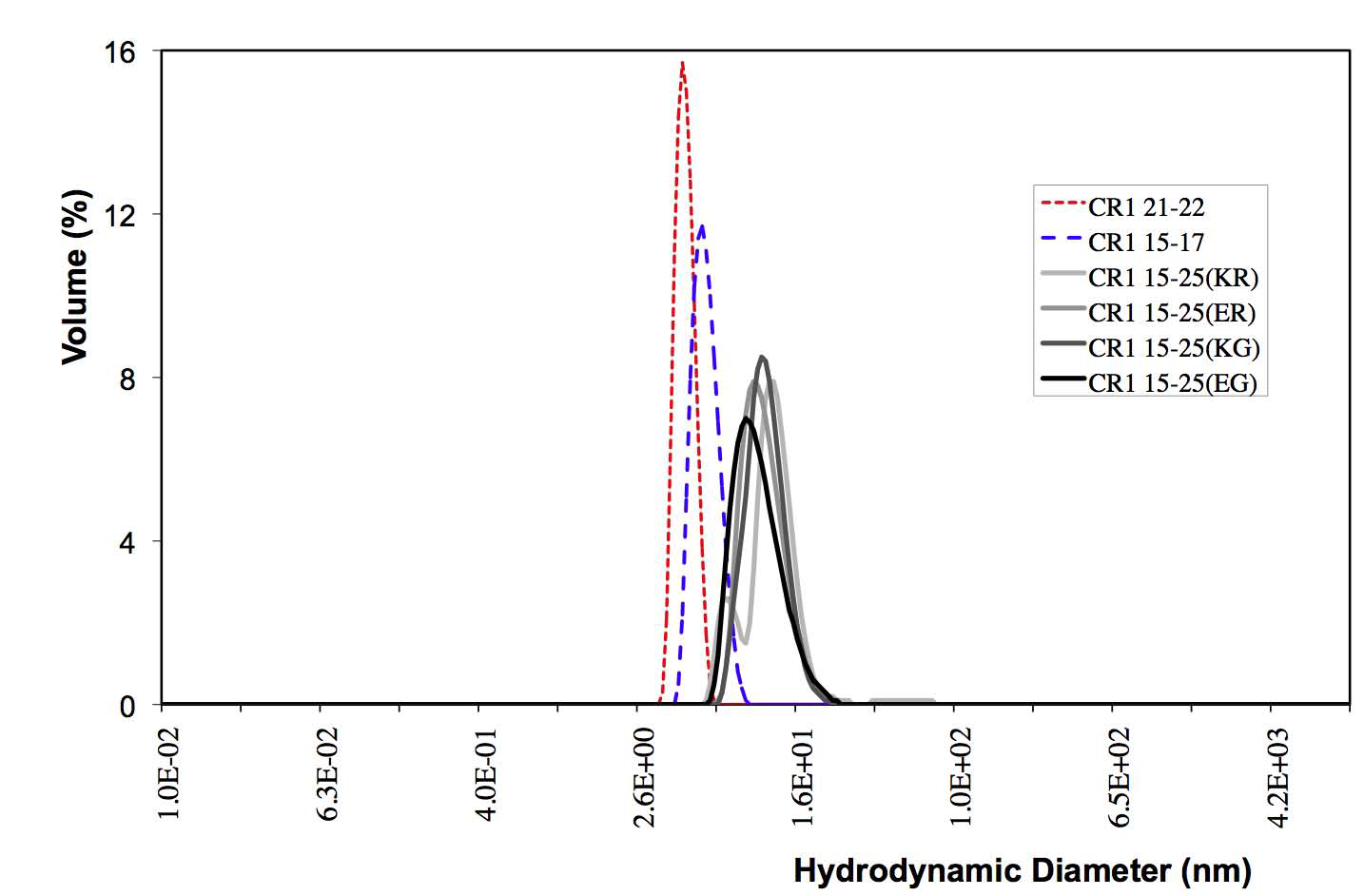

Supplement: Figure S1 — Particle size distribution according to dynamic light scattering. Shown are overlays of dynamic light scattering-derived particle size profiles for the indicated recombinant CR1 fragments (see key). Data were collected using a Zetasizer Nano S system (Malvern Instruments Ltd., UK) on samples of ∼3.5 mg.ml−1 protein in phosphate-buffered saline at 25°C. (TIFF) [file pone.0034820.s001.tiff]

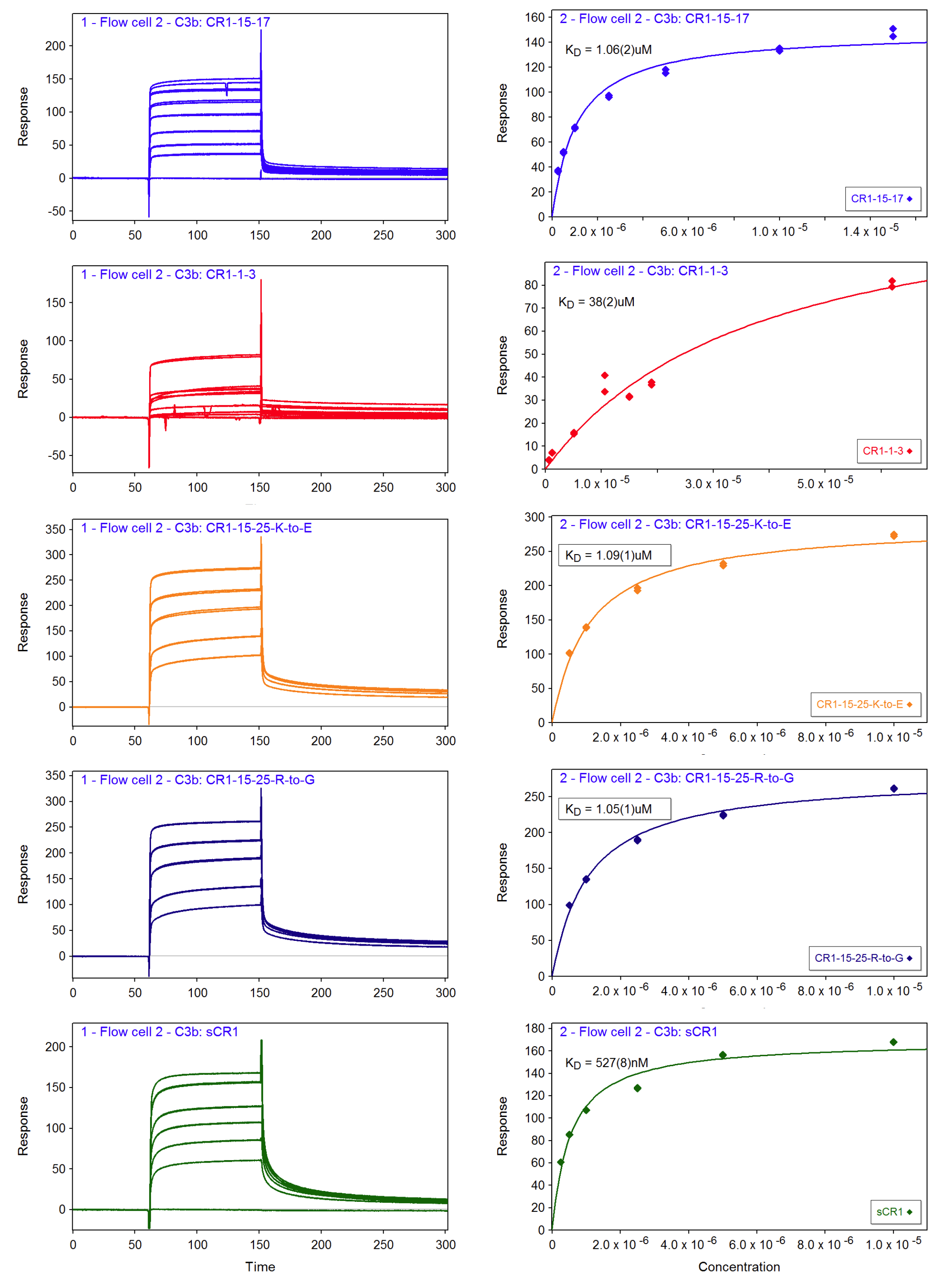

Supplement: Figure S2 — Binding of CR1 constructs to C3b by SPR. Sensorgrams (left) (for a concentration series, see Methods in main text) and response (response units (RU)) versus concentration (M) plots (right) for, from top to bottom, CR1 15–17 (site 2), CR1 1–3 (site 1), CR1 15–25 (ER), CR1 15–25 (KG) and sCR1. The fitted (see Methods in main text) K D values (shown in µM ± error, where the error applies to last significant figure shown, e.g. for CR1 15–17, K D = 1.06±0.01 µM) are displayed in the plots but also summarized in Table 2, main text. (TIFF) [file pone.0034820.s002.tiff]

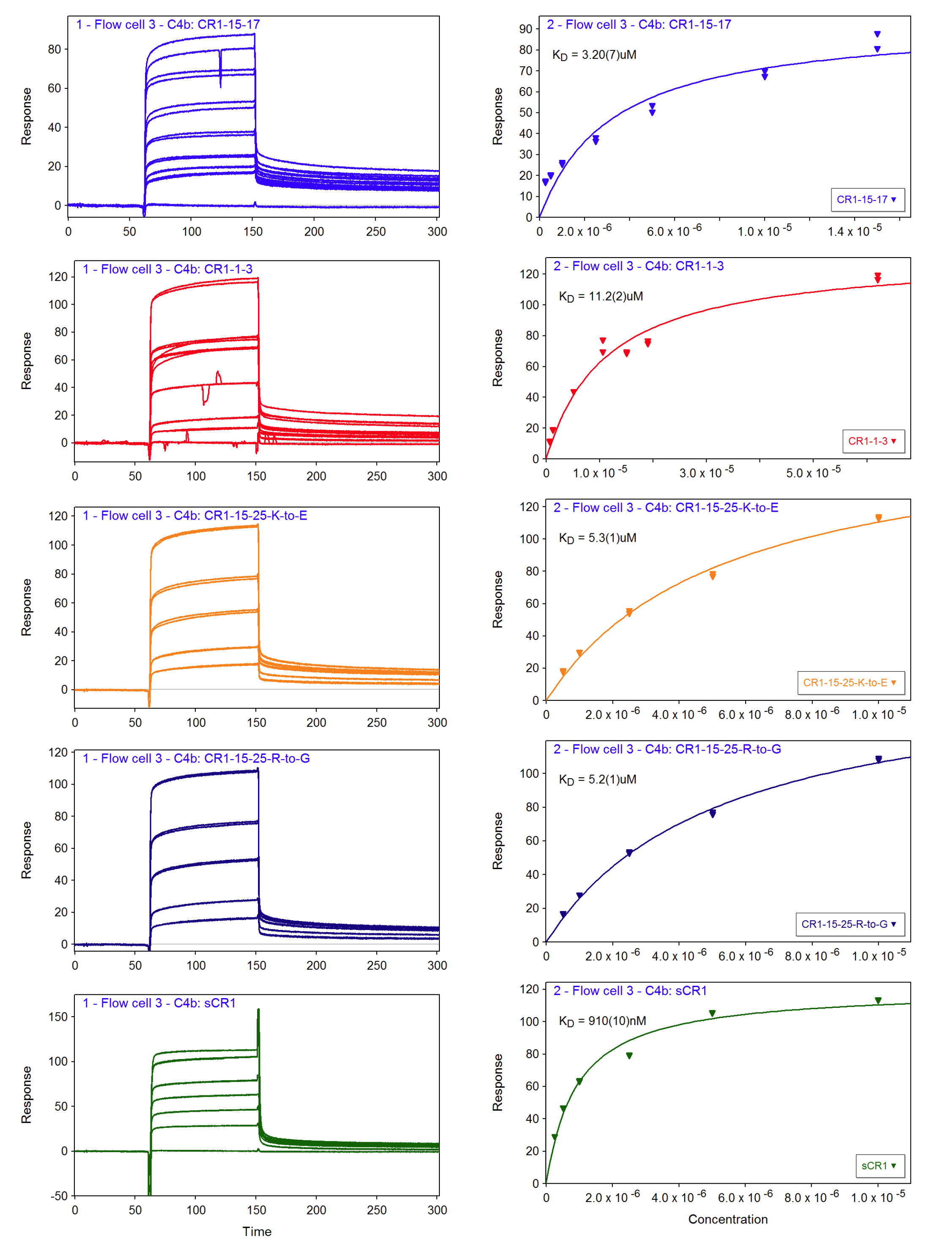

Supplement: Figure S3 — Binding of CR1 constructs to C4b by SPR. As for Figure S2 except C4b replaced C3b. (TIFF) [file pone.0034820.s003.tiff]

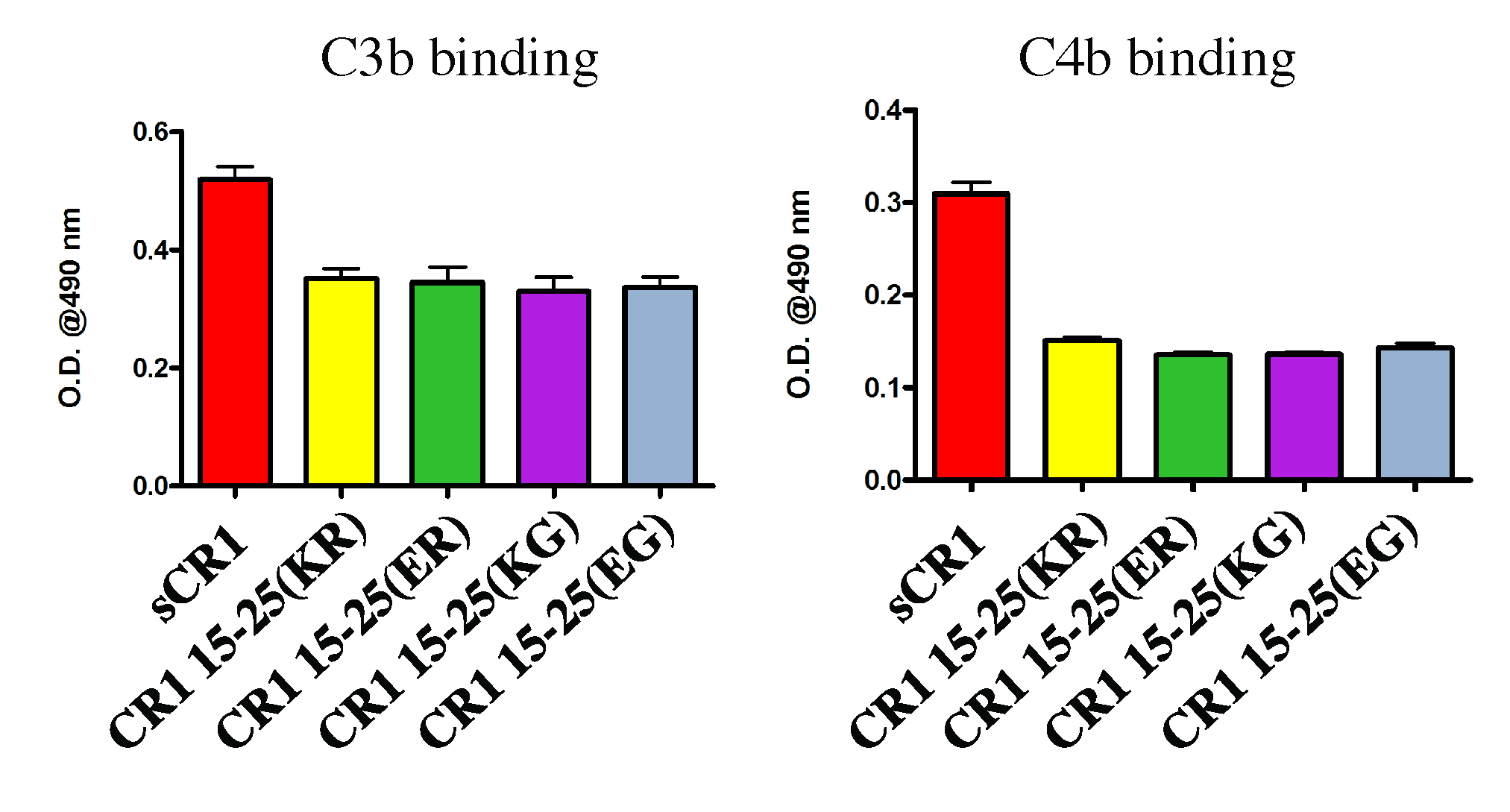

Supplement: Figure S4 — Binding of CR1 constructs to C3b and C4b by ELISA. There are no significant differences between the CR1 15–25 variants in terms of their ability to bind to C3b and C4b according to an ELISA (see Methods in main text). (TIFF) [file pone.0034820.s004.tiff]

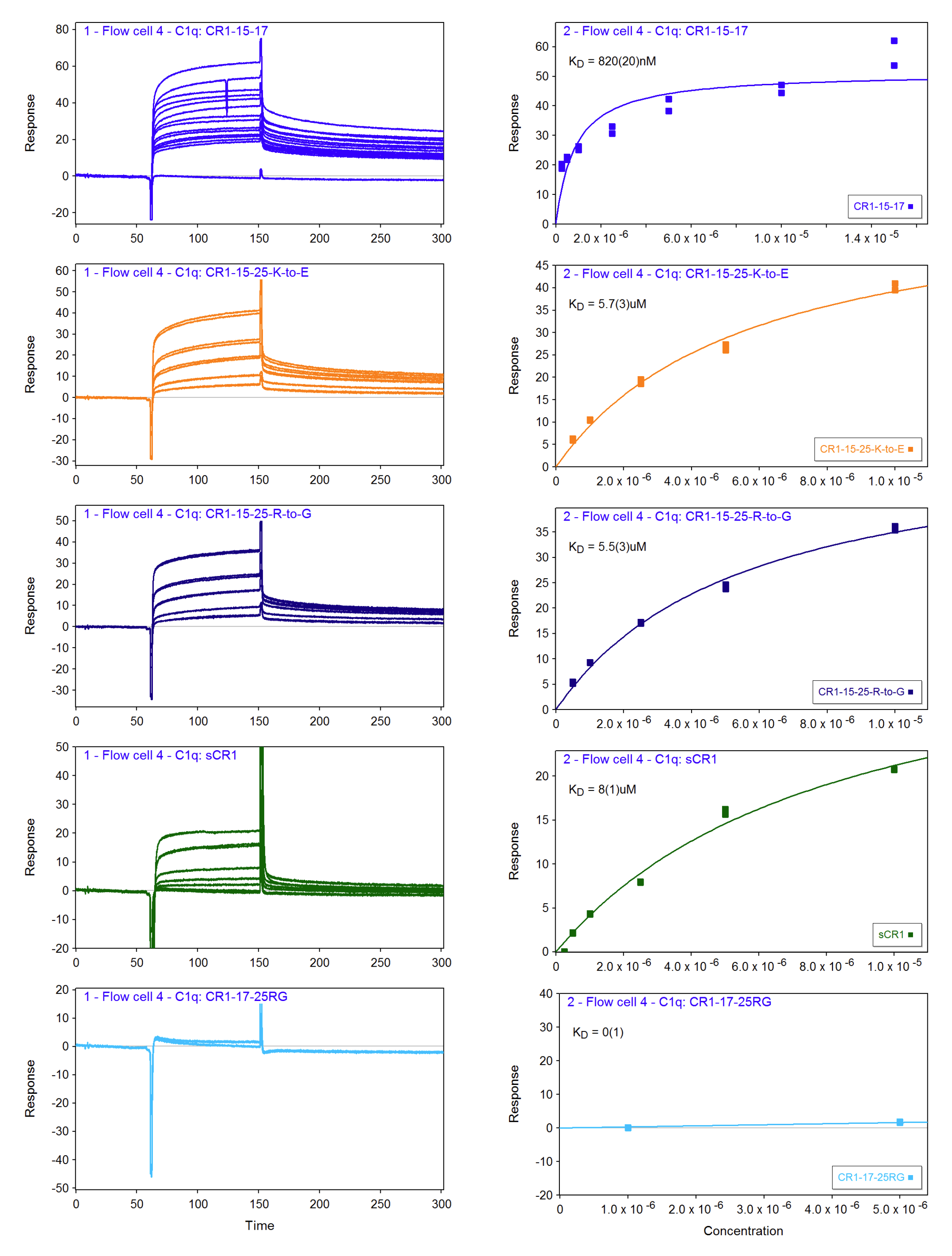

Supplement: Figure S5 — Binding of CR1 constructs to C1q by SPR. As for Figure S2 except C1q replaced C3b. (TIFF) [file pone.0034820.s005.tiff]
